# When prenatal infection meets postnatal hyperoxia: Better models for bronchopulmonary dysplasia and its therapeutic approaches

**DOI:** 10.1016/j.pccm.2024.11.002

**Published:** 2024-12-13

**Authors:** Ying Dong, Annika Leidner, Manuela Marega, Stefano Rivetti, Saverio Bellusci

**Affiliations:** a Department of General Pediatrics and Neonatology, Justus-Liebig-University, Giessen 35392, Germany; b Universities of Giessen and Marburg Lung Center, Cardio-Pulmonary Institute, Giessen 35392, Germany

To the editor,

With increased survival of extremely preterm infants (EPIs) born at 22–28 weeks’ gestational age (GA), bronchopulmonary dysplasia (BPD) has become one common and severe complication of EPI survivors.^1^ Despite abundant clinical research, there is a lack of strategies to effectively prevent or treat BPD, and preclinical animal models are indispensable tools to elucidate BPD pathogenesis and seek potential pharmaceutical therapies. Here, we introduce a novel two-hit rodent model incorporating both prenatal infection and postnatal hyperoxia which closely simulates BPD, providing an optimized preclinical platform for mechanistic and therapeutic research.

## Paradigm shift in BPD characteristics and rationale for better animal models

Due to advances in perinatal–neonatal healthcare especially in the post-surfactant era, the pathology of BPD has evolved from generalized airway and vascular damage with diffuse fibrosis (“old BPD”) to arrested lung development marked by simplified alveolarization and remodeling of vasculature (“new BPD”).^1^^,^^2^ Current knowledge of BPD development emphasizes its multifactorial origin spanning over pre-, peri-, and postnatal period.^2^ However, the majority of preclinical models use one risk factor, mostly postnatal hyperoxia, to reproduce BPD pathology as observed in EPIs. One-hit hyperoxia models failed to capture the multifactorial etiology of BPD, raising in the first place concerns of their clinical relevance.

Based on the fact that chorioamnionitis is the main driver of preterm delivery and occurs in up to 70% of EPIs, preclinical studies have increasingly included prenatal infection as the first hit to induce BPD.^1^^,^^3^ Due to the general replacement of tracheal intubation by non-invasive respiratory support in clinical practice, mechanical ventilation constitutes a much less significant risk factor for new BPD as for old BPD.^1^ The same applies to many other postnatal risk factors (e.g., repeated nosocomial infection and insufficient nutritional intake), thanks to progress in neonatal intensive care.^1^^,^^2^ Nonetheless, cumulative oxygen overexposure or hyperoxia still remains a significant contributor to new BPD, especially the severe form requiring long-term home oxygen therapy. As such, prenatal infection and postnatal hyperoxia stand out as dominant contributors of BPD in current clinical circumstances. So far, animals used for BPD models include rodents, rabbits, pigs, sheep, and non-human primates. Compared to other animals, rodents provide distinctive advantages especially broad possibilities of genetic modification, enabling detailed mechanistic and targeted therapeutic investigations. Moreover, rodent models have more rapid experimental turnout, relatively higher cost-effectiveness and less ethical considerations than other larger animals. Taken together, it is rational to use two-hit rodent models incorporating both prenatal infection and postnatal hyperoxia in the new BPD era.

## Characteristics of stimuli are clinically relevant in current two-hit rodent models

**Fig. 1:**
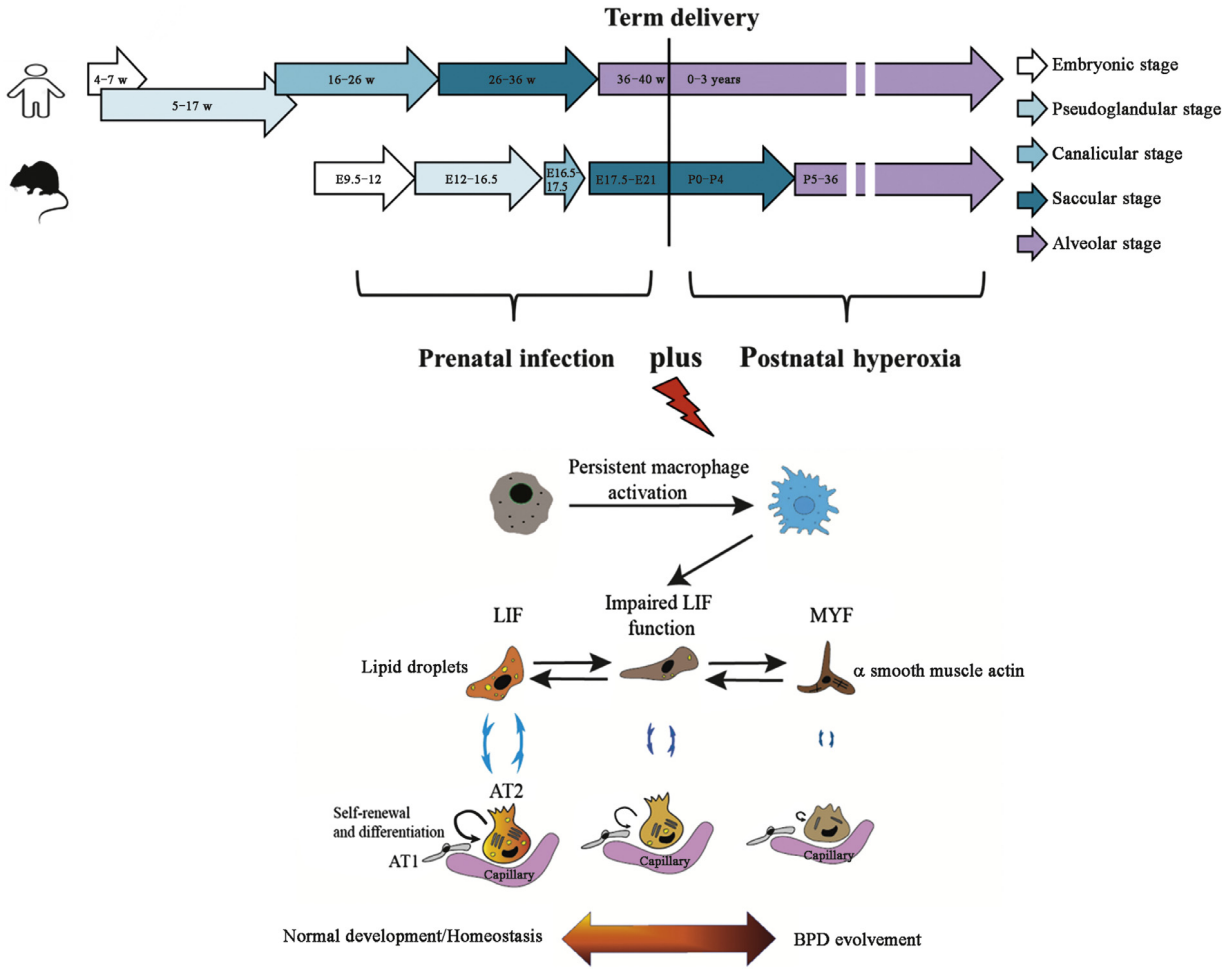
Schematic illustration of lung development in human and rodents and macrophage–mesenchyme crosstalk to drive BPD development. AT: Alveolar type; BPD: Bronchopulmonary dysplasia; E: Embryonal day; LIF: Lipofibroblast; MYF: Myofibroblast.

Prematurely delivered animals, regardless of species, have limited chance of *ex utero* survival. Rodents are born with lung development at the saccular stage (Fig. 1), which is structurally similar to that of human EPIs, thus providing the rationale for using rodents delivered at term to establish preclinical BPD models.^3^^,^^4^ Lipopolysaccharide (LPS) has been commonly applied to pregnant animals via an intra-peritoneal (i.p.) or intra-amniotic (i.a.) route, covering both systemic and localized prenatal infection as observed during the pregnancy.^5^, ^6^, ^7^, ^8^, ^9^ To mimic acute and prolonged duration, LPS was administered between the pseudoglandular and saccular stages.^5^, ^6^, ^7^, ^8^, ^9^ The doses of LPS varied from 0.5 µg/sac to 10 µg/sac i.a. and 50 µg/kg to 200 µg/kg i.p., respectively, reproducing different severities of prenatal infection.^3^^,^^5^, ^6^, ^7^, ^8^, ^9^ After birth, pups were subjected to hyperoxia ranging from 40% to ≥95% over a period of up to 4 weeks, with possibility to be returned to room air afterward to simulate the clinical recovery phase.^5^, ^6^, ^7^, ^8^, ^9^ In the end, molecular profiles of the lung, echocardiograph, lung microCT and histopathology, and pulmonary function test were performed to assess BPD phenotypes.^5^, ^6^, ^7^, ^8^, ^9^

## One- *vs.* two-hit rodent BPD models and their comparability with human BPD

Both one- and two-hit rodent models are able to mimic the molecular, morphological, and functional profile of BPD-like lung injuries, to a certain extent (Table 1).^5^, ^6^, ^7^, ^8^, ^9^ Prenatal infection and/or postnatal hyperoxia have been demonstrated to trigger proinflammatory cytokine reaction in the lung and sustain an activated status of immune cells, such as macrophages and T lymphocytes.^5^, ^6^, ^7^ Meanwhile, proteinases, growth factors, and vascularization-related mediators were dysregulated.^8^^,^^9^ These molecular changes are in alignment with BPD patient-derived biosample data.^2^ At the endpoint, animals display large and simplified alveoli with signs of pulmonary vascular remodeling, similar to new BPD of human EPIs (Table 1).^5^, ^6^, ^7^, ^8^, ^9^ Importantly, current rodent models are capable of recapitulating BPD long-term sequelae, demonstrating significant and persistent growth retardation from the first postnatal week to adulthood (Table 1).^5^, ^6^, ^7^^,^^9^ In addition, severe form of BPD exhibiting reduced right ventricle function, pulmonary hypertension, lung fibrosis, and decreased airway compliance was also observed in rodent models.^5^^,^^7^^,^^8^ However, not all hallmarks of human BPD can be well reproduced in rodents due to interspecies differences. In particular, the male disposition for BPD development in human was less likely to be generated in rodent models, and a much higher oxygen concentration >65% is generally required to induce lung injury in rodents due to their innate resistance to hyperoxia.^4^, ^5^, ^6^^,^^9^

**Table 1:** Representative rodent two-hit BPD models of both prenatal infection and postnatal hyperoxia.

Animals	Prenatal infection	Postnatal hyperoxia	Fetal/neonatal outcome	Molecular changes of the lung	Structural and functional changes of the lung
C3H/HeN mice^5^	i.p. LPS (0111:B4) 80 µg/kg at 16 dpc	85% HYX for 14 days and then RA for 14 days	20% of dams did not deliver after prenatal LPS. Pups’ weight at P1: Controls > LPS group; at 8 weeks: Control equals LPS alone > HYX alone > LPS + HYX	At P7 and P14: compared with either insult alone, LPS + HYX ↑ P-SMAD2, *Tnf, KC, Tgfb1, Il1b, Il6, Ccr2*, and *Col1a1*, ↓ *miR-29b.* At P14 and P28: LPS + HYX ↑ No. of macrophage *vs*. LPS alone	At P14 and P28: Compared with either insult alone, LPS + HYX ↓ alveolarization, ↑ collagen deposition, and impaired lung mechanics at P14, which persisted to P28 At 8 weeks: LPS + HYX ↑ lung expansion, alveolar simplification and interstitial/septal markings and lung density in CT *vs*. HYX or LPS alone
C57BL/6J mice^6^	i.p. LPS (0111:B4) 150 µg/kg at E14⁎	65% or 85% HYX for 28 days	Pups’ weight at P28: Controls > LPS + 65% HYX > LPS + 85% HYX	At P3: LPS + 85% HYX ↑ IL1, IL6, CXCL2 *vs*. controls, ↑ IL1B, C5a, CCL3, CXCL13 *vs*. LPS alone At P28: Compared to controls, LPS + HYX (both 85% and 65%) ↓ No. of CD45+ immune cells, but no effect on CD4+/CD8+ T cells, B cells, dendritic cells, or F4/80 + macrophage. LPS + HYX ↑ CD11b and Ly6G expression of macrophage *vs*. LPS alone	At P14 and P28: LPS + HYX ↓ alveolar number, ↑ alveolar size *vs*. LPS alone. The effect of LPS + 85% HYX > LPS + 65% HYX with increased lung injury at P28 than at P14
C57BL/6 mice^7^	i.p. LPS (055:B5) 200 µg/kg at 16 dpc	50% HYX for 7 days and then RA for 14 days	Pups’ weight at P1: Controls equals LPS group At P5: LPS + HYX ↓ weight *vs*. controls At P14: no difference among all groups At P21: LPS and LPS + HYX both ↓ weight *vs*. controls, no difference between LPS and LPS + HYX	At P1: LPS ↑ *Tlr4, Tnf, Il1b, Il6, Tgfb1 vs*. controls At P5: similar expression of genes above in LPS + HYX *vs*. HYX At P14: Compared to controls, HYX or LPS ↓ *Ctgf*. LPS + HYX showed no synergy *vs*. either insult alone At P21: LPS or HYX alone ↑ *Tgfb1, Col3a1,* and *Ctgf*. LPS + HYX showed no synergy *vs*. either insult alone	At P21: either LPS or HYX alone ↑ resistance of ASM, ↓ airway compliance *vs*. controls. LPS + HYX showed no synergy *vs*. either insult alone
C57BL/6 mice^8^	i.p. LPS (source unknown) 100 µg/kg at E18⁎	40% HYX for 7 days and then RA for 2 weeks	Spontaneous delivery 3 days after LPS	At P21: LPS alone or LPS + HYX ↑ *Nos2* and *Tgfb*, no synergy of LPS and HYX was observed. *Tnf, Ilb*, and *Il6* unaffected in LPS and/or HYX	At P21: airway and vessel reactivity in HYX alone > LPS + HYX > LPS alone equals controls
Sprague-Dawley rats^9^	i.a. 10 µg/sac LPS (055:B5) at 20 dpc	80% or ≥95% HYX for 14 days	C-section at 22 dpc Mortality at P1: LPS group 59%; 80% HYX group 97%; LPS + 80% HYX: 85% Pups’ weight at P14: LPS + 80% HYX >80% HYX >LPS alone	At P1 and P5: LPS ↓ VEGF and VEGFR2, LPS + 80% HYX ↑ VEGF and VEGFR2 *vs*. LPS alone	At P14: LPS or 95% HYX ↓ number of alveoli and vascular density, ↑ MLI and vascular wall thickness *vs*. controls. LPS + 80% HYX alleviated alveolar and vascular abnormalities *vs*. LPS alone but LPS + 95% HYX further aggravated lung damages *vs*. LPS alone

⁎ Pregnancy was not confirmed by plug check. ASM: Airway smooth muscle; BPD: Bronchopulmonary dysplasia; CCL: Chemokine (C-C motif) ligand3; CCR: Chemokine (C-C motif) receptor; Col1a1: Collagen type I alpha 1; Col3a1: Collagen type III alpha 1; CTGF: Connective tissue growth factor; CXCL: Chemokine (C-X-C motif) ligand; dpc: Day post coitum; E: Embryonal day; HYX: Hyperoxia; i.a.: Intra-amniotic; i.p.: Intra-peritoneal; IL: Interleukin; Ly6G: Lymphocyte antigen 6 family member G; KC: Keratinocyte-derived chemokine; LPS: Lipopolysaccharide; MLI: Mean linear intercept; NOS: Nitric oxide synthase; P: Postnatal day; RA: Room air; TGF: Transforming growth factor; TLR: Toll-like receptor; TNF: Tumor necrosis factor; VEGF: Vascular endothelial growth factor; VEGFR2: Vascular endothelial growth factor receptor 2.

Of note, prenatal LPS may increase the number of alveolar type 2 (AT2) cells and enhance the expression of surfactant proteins in rodent lungs.^3^ This is consistent with some clinical data showing reduced prevalence of respiratory distress syndrome in certain groups of EPIs exposed to chorioamnionitis.^1^ In contrast, maturity-promoting effect was not noticed for hyperoxia.^5^, ^6^, ^7^, ^8^, ^9^ Compared to one-hit models, a two-hit setting is able to dissect the interaction between various factors and their net effect on the immature lung. So far, complex real-life scenarios comprising prolonged or acute prenatal infection as well as postnatal moderate or severe hyperoxia have been explored in two-hit models.^5^, ^6^, ^7^, ^8^, ^9^ While some demonstrated synergistic interaction between LPS and hyperoxia to aggravate lung structural and functional impairment, others pointed toward marked alleviation of LPS-induced lung injury by subsequent oxygen exposure.^5^, ^6^, ^7^, ^8^, ^9^ It seems that lower concentration and shorter duration of postnatal hyperoxia may have the potential to antagonize the effect of prenatal LPS. Taken together, perinatal factors can exert both protective and injurious effects on the immature lung. Two-hit models more closely approximate the wide spectrum of clinical events that precipitate BPD, and may convey useful information for risk prediction of BPD based on the interaction of perinatal factors.

## Two-hit rodent models for exploring therapeutic alternatives for BPD

After decades of clinical research, systemic caffeine administration, postnatal low-dose corticosteroids, and intramuscular supplementation of vitamin A constitute the only pharmaceutical approaches with evidence-based efficacy and safety to treat BPD.^1^^,^^3^ Therefore, preclinical investigations are important to expand our therapeutic armamentarium for BPD. Since transcriptomic data have shown that LPS and hyperoxia trigger signaling transduction differentially in the developing lung,^2^^,^^3^^,^^10^ two-hit models should be preferred over one-hit models to deliver more comprehensive mechanistic understanding.

Based on recent single-cell RNA sequencing data, it seems that pulmonary macrophages are initial responders to various stimuli (Fig. 1), sending out signals to other lung compartments of mesenchyme, epithelium, and endothelium.^2^^,^^10^ Nowadays, the most premature infants delivered in the canalicular phase of lung development are able to survive but at the same time have the highest risk of BPD.^1^ During the very early stages of lung organogenesis, mesenchyme stands out as the central player, as they contribute to the niche of epithelial and endothelial stem cells and instruct their developmental behavior with increasing gestation. Pioneer studies with a single-cell view have identified lung mesenchyme to comprise highly heterogeneous subpopulations.^2^^,^^10^ Among them, lipofibroblasts (LIFs) and myofibroblasts (MYFs) characterized by the synthesis of lipids and alpha-smooth muscle actin (α-SMA), respectively, may undergo two-way conversion to modulate lung homeostasis and disease progression (Fig. 1).^10^ In particular, fibroblast growth factor 10 (FGF10) as a signature marker of LIFs has been shown to promote the maturity of surfactant-producing AT2 cells but was significantly reduced in tracheal aspirates and lung explants of BPD patients.^2^^,^^10^ The i.p. administration of recombinant FGF10 may trigger *de novo* alveologenesis in newborn mice and alleviate pre-existing BPD-like injuries by modulating LIFs–MYFs transition.^10^

In light of the above, dissecting macrophage–mesenchyme crosstalk and the consequent mesenchymal dynamics may shed light on potential druggable targets for BPD treatment. Notably, there is discordance between rodents and human regarding macrophage and mesenchymal markers and their ontogeny. Therefore, caution is needed in extrapolating preclinical data in patient-based investigations.

## Limitations of rodent two-hit models

First, a number of current models demonstrated a high rate of abortion and/or stillbirth following prenatal LPS exposure, ranging from 15% to 60%.^3^^,^^5^^,^^9^ This contradicts the clinical fact that the majority of preterm infants survive despite exposure to prenatal infections.^1^ Second, various rodent strains, doses, and durations of stimuli were used in the current research.^3^, ^4^, ^5^, ^6^, ^7^, ^8^, ^9^ This results in wide heterogeneity of BPD phenotypes across studies, rendering it difficult to draw clear conclusion on the impact of two-hit exposure on the immature lung. Reducing offspring mortality by optimized administration of injurious stimuli and standardization of methodology is warranted in future BPD models. Last but not the least, the interspecies difference between rodents and human in terms of physiological characteristics may restrict the translational utilization of preclinical models, especially when choosing transgenic mice for pathway analysis and testing particular pharmaceuticals.

## Conclusion and future outlook

BPD persists to be a great healthcare concern due to the growing population of EPIs who can now survive into adulthood. The frustration to significantly reduce BPD incidence and improve its treatment is mainly attributed to the multifactorial origin as well as complex patterns of disease development. Given the limited access to newborn lung biosamples and ethical difficulties in conducting clinical studies during the perinatal period, animal models are still relied on for an in-depth pathomechanistic understanding of BPD and the search of therapeutic options. A two-hit rodent model of prenatal infection followed by postnatal hyperoxia better simulates the clinical situation of most EPIs, thus being more advantageous for BPD research than single-hit models. Yet, challenges exist in standardizing multiple exposure factors across different studies and extrapolating rodent data to human patients due to physiological differences between species.

## Declaration of competing interests

The authors declare that they have no known competing financial interests or personal relationships that could have appeared to influence the work reported in this paper.
